# 

**Published:** 1828-11

**Authors:** 


### MONTHLY LIST OF MEDICAL BOOKS.

[*Medical Works cannot be entered on this List except a copy be sent for the purpose ; the titles of Books having frequently been transmitted to us, as published, which have not appeared for weeks, or even months, after.*]

Observations on the Nature and Treatment of Cholera; and on the Pathology of Mucous Membranes. By ALEX. TURNBULL CHRISTIE, M.D.—Maclachlan and Stewart, Edinburgh, 1828.

A Manual of the Anatomy, Physiology, and Diseases of the Eye and its Appendages. By S. J. STRATFORD.—Longman, London, 1828.

An Essay on a new Mode of Treatment of Diseased Joints, and the Non-Union of Fracture; with Cases and Formulæ of the various Preparations used. By THOMAS BUCHANAN, C.M.—Longman, London, 1828.

A Manual on Midwifery; or, a Summary of the Science and Art of Obstetric Medicine: including the Anatomy, Physiology, Pathology, and Therapeutics, peculiar to Females; Treatment of Parturition, Puerperal and Infantile Diseases, &c. By MICHAEL RYAN, M.D.—Longman, London, 1828.

Observations on the Nature and Treatment of Fractures of the upper Third of the Thigh-bone, and of Fractures of long standing, &c. &c. By JOSEPH AMESBURY.—T. and G. Underwood, London, 1828.

### NOTICES.

*Communications to be addressed* (post paid) *to the Publisher.*

